# Prediction of incident heart failure in men and women with a history of myocardial infarction

**DOI:** 10.1093/eschf/xvag187

**Published:** 2026-06-30

**Authors:** Phoebe Chan, Thomas F Kok, Robert M A van der Boon, Navin Suthahar, Rudolf A de Boer, Eric Boersma, Isabella Kardys

**Affiliations:** Department of Cardiology, Erasmus MC, Cardiovascular Institute, Thorax Centre, P.O. Box 2040, Rotterdam 3000 CA, The Netherlands; Department of Cardiology, Erasmus MC, Cardiovascular Institute, Thorax Centre, P.O. Box 2040, Rotterdam 3000 CA, The Netherlands; Department of Cardiology, Erasmus MC, Cardiovascular Institute, Thorax Centre, P.O. Box 2040, Rotterdam 3000 CA, The Netherlands; Department of Cardiology, Erasmus MC, Cardiovascular Institute, Thorax Centre, P.O. Box 2040, Rotterdam 3000 CA, The Netherlands; Department of Cardiology, Erasmus MC, Cardiovascular Institute, Thorax Centre, P.O. Box 2040, Rotterdam 3000 CA, The Netherlands; Department of Cardiology, Erasmus MC, Cardiovascular Institute, Thorax Centre, P.O. Box 2040, Rotterdam 3000 CA, The Netherlands; Department of Cardiology, Erasmus MC, Cardiovascular Institute, Thorax Centre, P.O. Box 2040, Rotterdam 3000 CA, The Netherlands

**Keywords:** Incident heart failure, Myocardial infarction, General population, Risk factors, Biomarkers

## Abstract

**Introduction:**

Individuals with prior myocardial infarction (MI) show increased risk of heart failure (HF), yet current risk prediction models leave room for improvement. We aimed to develop and validate a clinically viable prediction model for incident HF in persons with prevalent MI in a general population, and explore sex- and MI subtype-specific differences.

**Methods:**

We analysed UK Biobank participants with prior MI but absence of HF at baseline. Sociodemographic factors, clinical variables, and 59 blood biomarkers were included. The primary endpoint was the first in-hospital HF diagnosis. Backward selected Cox proportional hazards models were used to identify independent predictors of incident HF. Model performance was assessed via discrimination (C-index), using internal- and hold-out validation, and calibration.

**Results:**

A total of 4743 participants with prevalent MI; 81.4% male, median (P25–P75) age 62 (58–66) years, were included. During a median follow-up of 12.1 (11.1–13.0) years, 767 (16.2%) developed HF. Sixteen independent predictors were identified and eleven remained significant (*P* < .05) after treating all-cause mortality as a competing risk. These included several well-established predictors (age, body mass index, smoking, atrial fibrillation, diabetes) as well as haemoglobin [HR per 1 standard deviation (SD) increase: 0.88 (95% CI: 0.79–0.96)], mean reticulocyte volume {HR: [1.13 (95% CI: 1.03–1.23)], and neutrophil percentage [HR: 1.12 (95% CI: 1.03–1.21)], monocyte count [HR:1.11 (95% CI, 1.06–1.16)]. A trend towards an interaction (*P* < .1) between sex and MI subtype was present. Model discrimination was modest (C-index = 0.67) and calibration was adequate.

**Conclusion:**

Our study identifies several clinically accessible blood biomarkers as important risk factors for HF in post-MI individuals, and suggests interactions between sex and MI subtype. Further model refinement and external validation are needed.

## Graphical Abstract

**Graphical Abstract:**
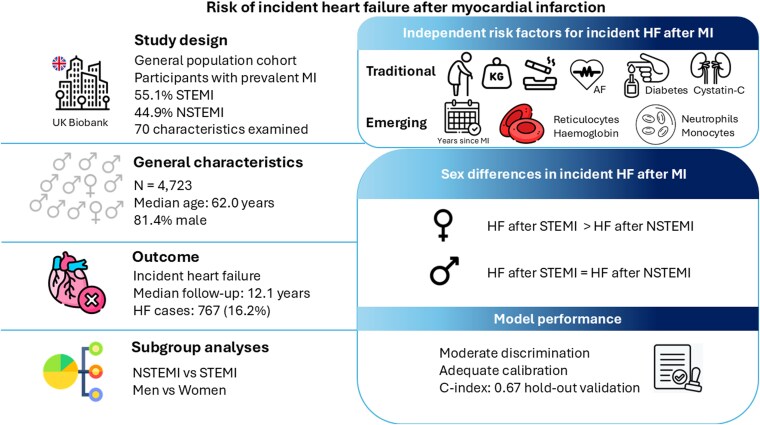

Overview of the investigation into incident heart failure (HF) risk among 4,723 post myocardial infarction (MI) individuals from the prospective UK Biobank study. The investigation resulted in a HF risk prediction model for post-MI individuals, featuring both traditional and emerging risk factors, with moderate discrimination, calibration and internal validation. Additionally, subgroup analyses revealed suggestive interactions between sex and MI-subtype.

## Introduction

Heart failure (HF) is a major health burden estimated to affect 56 million individuals worldwide.^1^ Myocardial infarction (MI) is an important precursor to HF;^2^ data from the Cardiovascular disease in Norway (CVDNOR) study found that 13% of participants who experienced acute MI, developed HF within 30 days after MI diagnosis, and an additional 33% between 30 days and 1 year after MI discharge.^3^ Patients with a history of MI who go on to develop incident HF have significantly worse prognosis, including higher rates of rehospitalization and mortality, compared with MI patients who remain free of HF.^4^

To mitigate the adverse consequences of HF, effective risk stratification in patients with a history of MI is essential. As the factors contributing to HF development vary among individuals with MI, a deeper understanding is needed to accurately assess risk and identify those at high risk for post-MI HF. Identifying high-risk individuals is crucial for guiding preventive strategies improving long-term outcomes through closer monitoring.^5^

Several studies have explored incidence and predictors of HF in patients with prevalent MI, although these were mostly based on clinical trial or hospital settings, featured short follow-up times and had limited validation, thus restricting their generalizability.^6–8^ Additionally, these models often failed to incorporate a comprehensive set of cardiovascular biomarkers or to adequately account for variations in MI subtypes or sex. As such, the long-term risk of incident HF after MI in the general population remains poorly characterized, which underscores the need for an accurate and validated risk prediction model for HF in a post-MI general population cohort.

In this study, we aimed to identify risk factors associated with the development of HF following MI. Furthermore, we aimed to develop and validate a comprehensive and accurate prediction model for incident HF in men and women with a history of MI. We pay particular attention to the predictive value of blood biomarkers and explore potential differences in MI subtypes and sex.

## Methods

### Study design and population

We used data from the UK Biobank, a cohort comprising 500 000 individuals aged 37 and over who underwent baseline assessments between March 2006 and July 2010. In the period following baseline, data on health, as well as environmental and lifestyle factors, were collected through health linkages and questionnaires. Written consent was provided by participants prior to enrolment.

In the current study, we included participants with clinically confirmed MI at baseline, who were identified using the *International Classification of Diseases 10th revision* (ICD-10) codes I21 and I22. Subjects with a documented history of HF (ICD-10 code I50) at the baseline visit were excluded.

### Data collection and variable definitions

In the UK biobank, baseline data was collected on lifestyle factors, sociodemographic factors, medical history, medication use, physical examination, and an extensive set of biomarkers. In total, 76 risk factors were included in this study; which was low enough to prevent model overfitting based on the number of reported primary outcome events (i.e. one-in-ten rule). Baseline lifestyle factors included smoking status (never, former, current), alcohol consumption frequency (daily, weekly, monthly, special occasions, never), and physical activity level (low, moderate, high), according to the International Physical Activity Questionnaire (IPAQ).^9^ Employment status was categorized as ‘in paid employment’, ‘retired’, or ‘other’.

MI subtypes were classified using ECG data as either non-ST-elevation MI (NSTEMI) or ST-elevation MI (STEMI). Cardiovascular and non-cardiovascular comorbidities, including atrial fibrillation (AF), hypertension, stroke, diabetes mellitus, chronic kidney disease (CKD), were confirmed by ICD-10 codes. Furthermore, medical history of four types of malignant cancers (breast, lung, colorectal, and haematological) were also included. Because detailed data on anticancer therapy were unavailable, a prior cancer diagnosis was used as a proxy, assuming all affected participants had received treatment. A complete list of used ICD-10 codes is provided in Supplementary Table S1.

Baseline use of antihypertensive, antidiabetic, and lipid lowering medication, as well as hormone replacement therapy (HRT) and oral contraceptives only applicable in women, were self-reported. Finally, we included 59 blood biomarkers, encompassing known biochemistry markers of overall disease expression or diagnostic markers, as well as blood count variables.

For any variable, the responses ‘do not know’ or ‘prefer not to answer’ were treated as missing. An overview of all variables, other than medical history variables, is shown in Supplementary Table S2.

### Primary outcome measure

The primary outcome was incident ICD10 in-hospital diagnosis of I50 (congestive HF, left ventricular failure, and unspecified HF). Survival time was the time in days between the date of UK Biobank assessment visit and an in-hospital diagnosis of incident HF event or, for patients who did not develop HF, time until the reported day of censoring. Participants were censored at the end of the follow-up period (September 2021), date of mortality, or when consent was withdrawn.

For the secondary analysis using competing risk regression, mortality was treated as a competing risk event rather than censored.

### Statistical analysis

Normally distributed continuous variables are presented as mean ± standard deviation (SD), and non-normally distributed variables as median (25th–75th) percentile. Categorical variables are summarized as counts and percentages. Differences in baseline characteristics between sexes were assessed using the two-sample *t*-test or Wilcoxon rank-sum test for continuous variables, depending on data distribution, and the χ^2^ test for categorical data. Correlations between baseline variables were evaluated using Spearman correlation, to prevent collinearity in the models that were subsequently fitted. In the case of collinearity, the most clinically relevant variable was selected. Continuous biomarker variables were standardized before they were entered into the models.

Cumulative incidence curves of HF were stratified by MI type (NSTEMI and STEMI) and by sex; incidence curves were generated using the Kaplan-Meier method. Differences between groups were evaluated using the log-rank test. Incidence rates of HF (per 1000 person-years) were calculated over the entire follow-up period. The effects of sex and MI type were further explored via interaction analyses.

To further assess the risk of incident HF development after MI in relation to potential baseline predictors, Cox proportional hazards (PH) models were used. Both univariable and multivariable Cox models were fitted, and PH assumptions were tested using Schoenfeld residuals. A backward selection procedure was applied to identify predictors for the final multivariable Cox model, retaining variables with *P* < .10. Sex was forced into the final model regardless of statistical significance, based on substantial evidence of sex-related differences in cardiac morphology and the clinical course of MI.^10^ As a secondary analysis, we conducted a competing risk analysis of HF, treating ACM as a competing event, using the Fine-Gray model.

The full dataset was split in a train (80%) set and a hold-out (20%) set through stratified sampling, which ensured similar proportions of HF within each set. The train set was further split into stratified five-fold cross-validation sets to assess internal model performance; which was defined as the average performance of the five models generated, iteratively, using data from four train folds, tested on a fifth fold. Multiple imputation by chained equations (MICE), using five iterations, was used to impute missing data; continuous variables were imputed via predictive mean matching while categorical variables were imputed via polytomous logistic regression. To prevent data leakage between train- and hold-out sets, each set was imputed separately. Also, to prevent data leakage during internal model validation, train data was split in five stratified folds of which, iteratively, four folds were combined and imputed (train set) while the fifth was imputed separately (test set). Information regarding missing data is shown in Supplementary Table S3.

Discriminative ability was evaluated in the full train data, in five-fold internal cross-validated train- and test sets, and in the hold-out set, using Harrell’s concordance index. C-indices were also optimism-corrected via the .632 estimator.^11^ Calibration was evaluated numerically via the Grønnesby and Borgan (GB) test statistic for four risk groups, and graphically by a calibration curve plotting predicted risks against observed HF rates at 10 years in the hold-out set using restricted cubic splines smoothing. The model was recalibrated by updating the baseline hazard on the holdout data.

Also, to demonstrate the potential clinical usefulness of our model, we compared C-indices between our final risk model and the well-established Pooled Cohort Equations to Prevent Heart Failure (PCP-HF) model^12^ at 10 years of follow-up. We also compared Cox models, one containing variables defined in the final model and the other containing variables in the PCP-HF score, via net reclassification improvement (NRI), which we defined as categorical NRI improvement for incident HF risk after 10 years of follow-up using risk categories: ‘0%–10%’, ‘10%–20%’ and ‘>20%’.

We report our findings as hazard ratios (HRs) and corresponding 95% confidence intervals (CI). Statistical analyses were conducted using R (4.3.2), using the packages mice, survival, rms, nricens, and tidycmprsk. All tests were two-tailed, and *P* values <.05 denoted statistical significance.

## Results

### Baseline characteristics and follow-up

**Figure 1:**
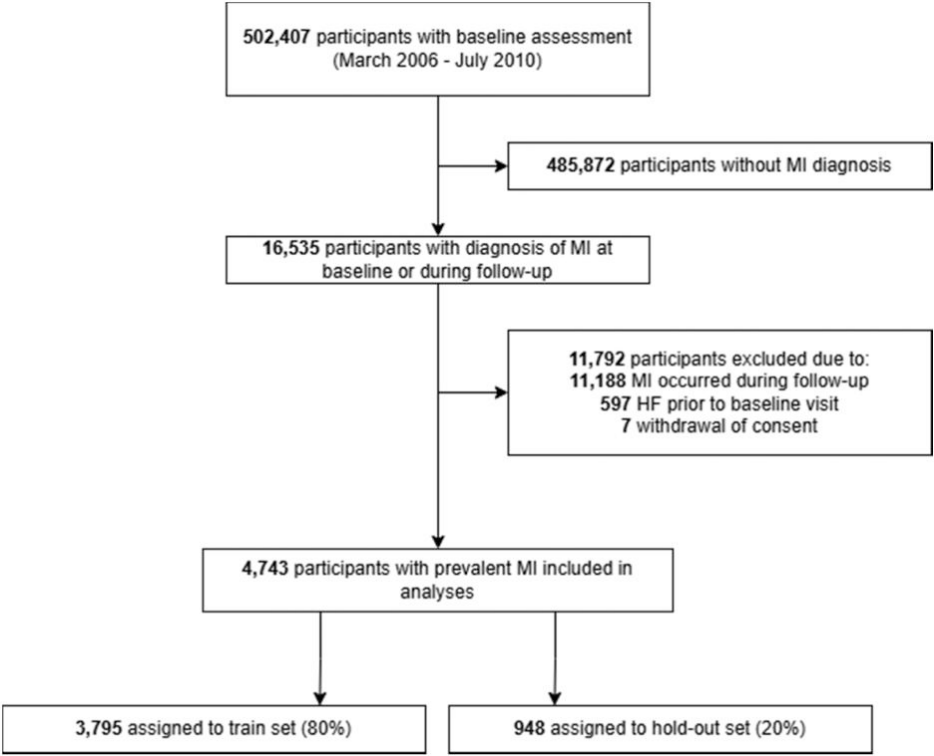
Flowchart of participant inclusion and subsequent splitting of data in train- and hold-out sets. HF, heart failure; MI, myocardial infarction

A total of 4743 participants with prevalent MI, were included in the study cohort; participant selection is shown in *Figure 1*. Baseline characteristics, stratified by sex, are described in *Table 1*. The median age of participants was 62 (IQR 58–66) years and 81.4% were men. The median time from MI onset to the baseline visit was 4.9 (IQR 2.3–7.9) years, with a significant longer duration in men [5.0 (2.3–8.0) years] than in women [4.4 (2.1–7.4) years]. At baseline, the most common risk factors and comorbidities included dyslipidaemias (50.0%), hypertension (47.0%), diabetes mellitus (11.7%), AF (7.5%), peripheral artery disease (PAD) (3.7%), obesity (3.2%), and CKD (0.6%). Overall, 55.1% of MIs were classified as STEMI, with a higher proportion in men (56.7%) than in women (47.9%). Men were more often former smokers and more likely to use cholesterol lowering medication and blood pressure medication compared with women. Laboratory values showed higher haemoglobin, creatinine and triglycerides levels in men, while total cholesterol levels were lower. Baseline characteristics, stratified by MI type, are further detailed in Supplementary Table S4.

**Table 1:** Baseline characteristics of the study population stratified by sex

	Valid cases	All, *n* = 4743	Women, *n* = 883	Men, *n* = 3860	*P* value
*Demographic characteristics*					
Age, y	4743 (100%)	62 (58–66)	63 (58–66)	62 (58–66)	.018
Ethnicity	4715 (99%)				.001
White		4459 (94.6%)	838 (95.3%)	3621 (94.4%)	
Asian or Asian British		168 (3.6%)	17 (1.9%)	151 (3.9%)	
Black or Black British		32 (0.7%)	12 (1.4%)	20 (0.5%)	
Mixed/other		56 (1.2%)	12 (1.4%)	44 (1.1%)	
Body mass index, kg/m^2^	4701 (99%)	28.4 (25.9–31.5)	28.3 (25.0–32.2)	28.4 (26.0–31.4)	.16
Systolic blood pressure, mm Hg	4456 (94%)	135 (124–149)	134 (122–149)	136 (124–150)	.18
Type of MI	4743 (100%)				<.001
NSTEMI		2130 (44.9%)	460 (52.1%)	1670 (43.3%)	
STEMI		2613 (55.1%)	423 (47.9%)	2190 (56.7%)	
Time between MI and baseline, years	4743 (100%)	4.9 (2.3–7.9)	4.4 (2.1–7.4)	5.0 (2.3–8.0)	.003
*Medical history*					
Atrial fibrillation	4743 (100%)	355 (7.5%)	62 (7.0%)	293 (7.6%)	.56
Chronic kidney disease	4743 (100%)	27 (0.6%)	5 (0.6%)	22 (0.6%)	.99
Diabetes mellitus	4743 (100%)	554 (11.7%)	91 (10.3%)	463 (12.0%)	.16
Dyslipidaemias	4743 (100%)	2372 (50.0%)	446 (50.5%)	1926 (49.9%)	.74
Obesity	4743 (100%)	154 (3.2%)	31 (3.5%)	123 (3.2%)	.62
Peripheral artery disease	4743 (100%)	175 (3.7%)	34 (3.9%)	141 (3.7%)	.78
Hypertension	4743 (100%)	2231 (47.0%)	434 (49.2%)	1797 (46.6%)	.16
Stroke	4743 (100%)	91 (1.9%)	23 (2.6%)	68 (1.8%)	.10
Cancer	4743 (100%)				
Breast cancer		29 (0.6%)	28 (3.2%)	1 (0.0%)	
Colorectal cancer		27 (0.6%)	5 (0.6%)	22 (0.6%)	.99
Haematologic cancer		31 (0.7%)	5 (0.6%)	26 (0.7%)	.72
Lung cancer		5 (0.1%)	1 (0.1%)	4 (0.1%)	>.99
*Lifestyle factors*					
Alcohol intake frequency	4727 (100%)				.15
Daily or almost daily		965 (20.4%)	191 (21.7%)	774 (20.1%)	
Three or four times a week		1085 (23.0%)	209 (23.7%)	876 (22.8%)	
Once or twice a week		1165 (24.6%)	204 (23.2%)	961 (25.0%)	
One to three times a month		547 (11.6%)	107 (12.1%)	440 (11.4%)	
Special occasions only		575 (12.2%)	88 (10.0%)	487 (12.7%)	
Never		390 (8.3%)	82 (9.3%)	308 (8.0%)	
Smoking	4705 (99%)				.001
Never		1534 (32.6%)	330 (37.7%)	1204 (31.4%)	
Former		2490 (52.9%)	420 (48.0%)	2070 (54.0%)	
Current		681 (14.5%)	125 (14.3%)	556 (14.5%)	
Physical activity	3782 (80%)				.18
Low		800 (21.2%)	151 (23.4%)	649 (20.7%)	
Moderate		1506 (39.8%)	261 (40.4%)	1245 (39.7%)	
High		1476 (39.0%)	234 (36.2%)	1242 (39.6%)	
*Medication use*	4675 (99%)				
Cholesterol lowering medication		4400 (92.8%)	797 (90.3%)	3603 (93.3%)	.001
Blood pressure medication		3626 (76.4%)	641 (72.6%)	2985 (77.3%)	.003
Insulin		200 (4.2%)	40 (4.5%)	160 (4.1%)	.61
Hormone replacement therapy^a^		32 (0.7%)	32 (3.6%)	NA	
Oral contraceptive^a^		5 (0.1%)	5 (0.6%)	NA	
No medication use		115 (2.4%)	38 (4.3%)	77 (2.0%)	<.001
*Laboratory results*					
Haemoglobin, g/dL	4545 (96%)	14.6 (13.8–15.3)	13.5 (12.8–14.1)	14.8 (14.1–15.5)	<.001
Creatinine, µmol/L	4433 (93%)	80.6 (70.8–91.4)	67.0 (59.4–76.0)	83.3 (74.5–93.8)	<.001
Cholesterol, mmol/L	4436 (94%)	4.2 (3.7–4.8)	4.6 (4.0–5.1)	4.2 (3.6–4.7)	<.001
Triglycerides, mmol/L	4430 (93%)	1.6 (1.1–2.3)	1.5 (1.1–2.1)	1.7 (1.2–2.4)	<.001

Values are reported as *n* (%), otherwise as median (Q1–Q3).

MI, myocardial infarction; NSTEMI, non-ST-elevation MI; STEMI, ST-elevation MI.

^a^Applicable to women only.

During a median follow-up of 12.1 years (IQR 11.1–13.0), 767 (16.2%) participants developed incident HF and 944 (20.6%) participants died; 302 (32.0%) deaths occurred in individuals with incident HF. The median time to HF diagnosis after the index MI was 12.5 years (IQR 9.6–16.3). The overall incidence rate of HF was 14.7 cases per 1000 person-years (95% CI 13.7–15.8) over the entire follow-up period.

### Sex and MI subtype differences

**Figure 2:**
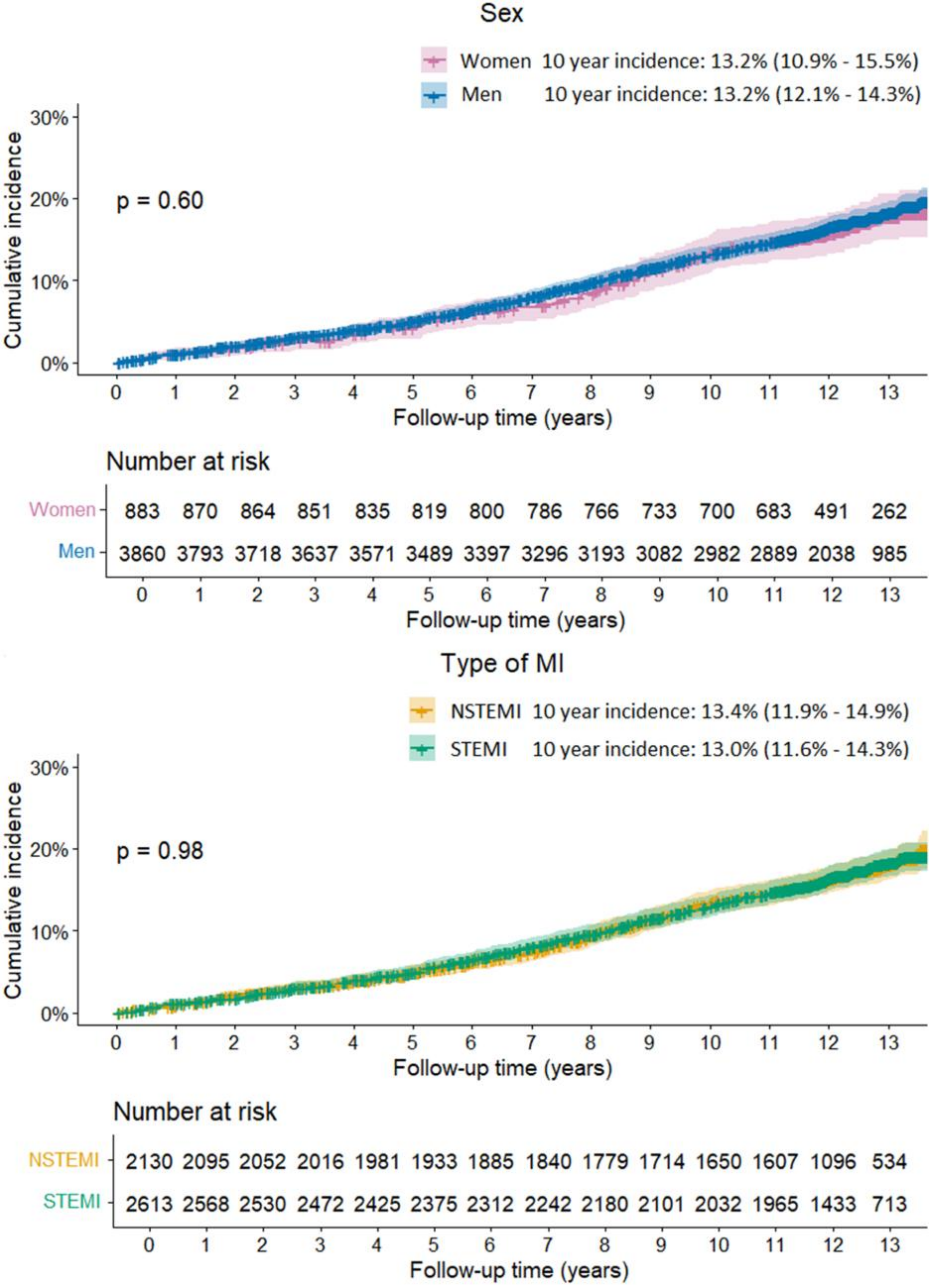
Cumulative incidence of heart failure with 95% confidence intervals in post-MI individuals, stratified by sex (upper figure) and by MI-subtype (lower figure). Survival time is given in years

**Figure 3:**
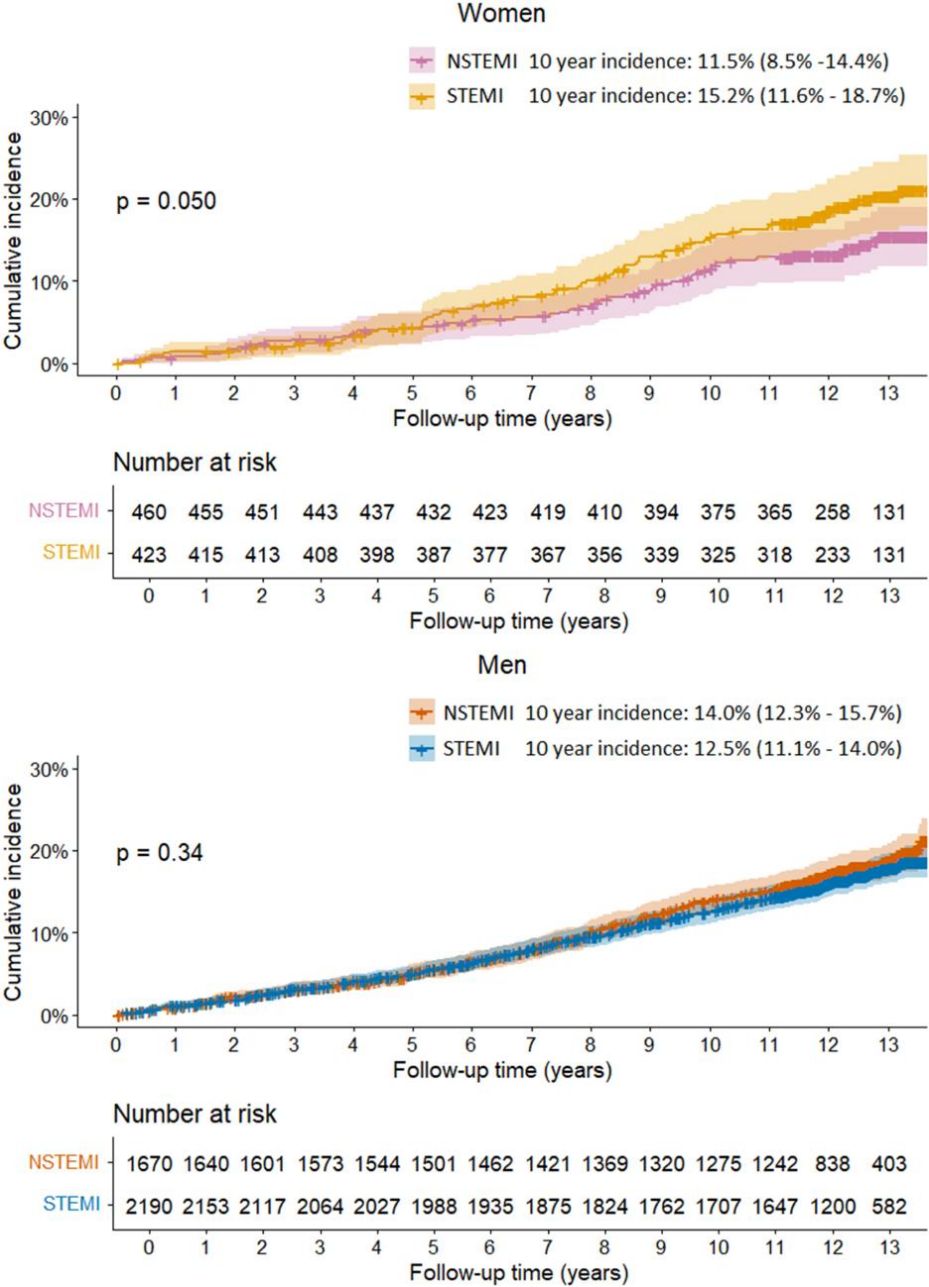
Cumulative incidence of heart failure with 95% confidence intervals in post-MI women (upper figure) and post-MI men (lower figure), stratified by MI-subtype. Survival time is given in years

**Figure 4:**
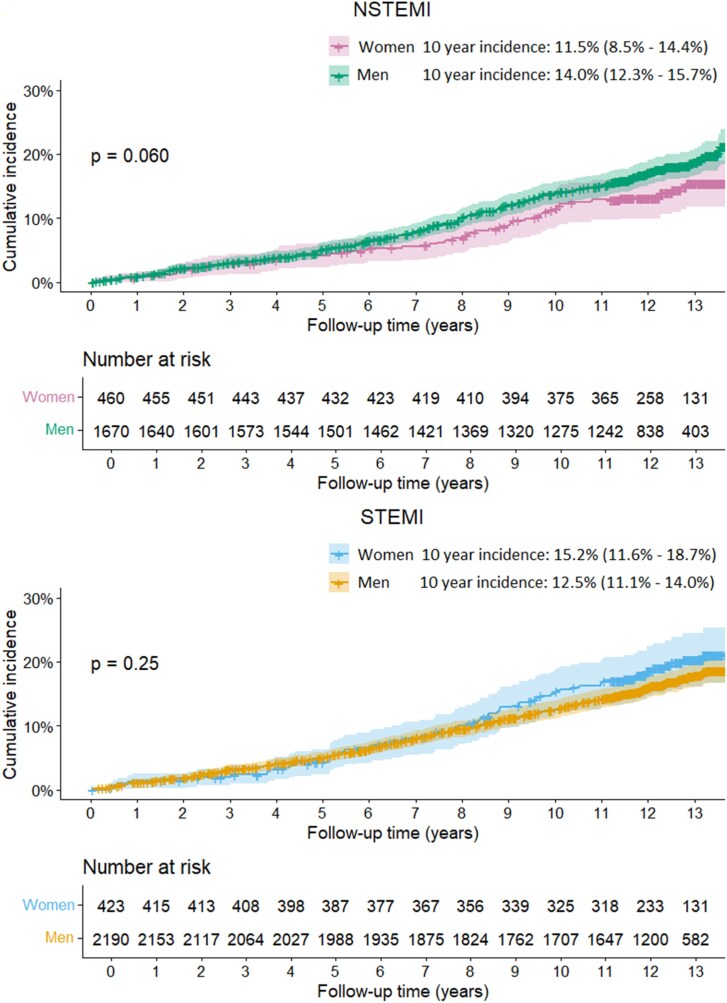
Cumulative incidence of heart failure with 95% confidence intervals in post-NSTEMI (upper figure) and post-STEMI (lower figure) individuals, stratified by sex. Survival time is given in years

There were no significant differences in incidence rates by sex (14.2 vs 14.9 per 1000 person-years for women and men, respectively; *P* = .60) nor by MI subtypes (14.8 vs 14.7 per 1000 person-years for STEMI and NSTEMI; *P* = .98) (*Figure 2*). Regarding the type of MI, women with STEMI had a higher incidence of HF than those with NSTEMI (16.7 for women with STEMI vs 12.0 per 1000 person-years for women with NSTEMI; *P* = .050), but no difference was observed among men (*Figure 3*). Among individuals with NSTEMI, men tended to have a higher incidence rate of HF than women (15.5 for men with NSTEMI vs 12.0 per 1000 person-years for women with NSTEMI; *P* = .060). In contrast, there was no significant difference among those with STEMI (14.4 for men with STEMI vs 16.7 per 1000 person-years for women with STEMI; *P* = .25) (*Figure 4*). Additionally, a trend (*P* < .1) was observed towards an interaction between sex and MI subtypes on the risk of incident HF (*P* = .095).

### Predictors of incident HF

The study cohort was split into a train (*N* = 3795) and hold-out set (*N* = 948) using stratified endpoint sampling. The train set was used for internal validation, while the hold-out set was used for hold-out set validation, and thus left out of the model creation process.

Multivariable Cox PH analysis identified 15 independent predictors of incident HF post-MI (*Table 2*). Sex was forced into the final model based on evidence from prior studies, resulting in sixteen independent predictors of HF. The PH assumptions were checked and satisfied.

**Table 2:** Associations between baseline risk factors and incident HF

Variable	Univariable Cox PH			Multivariable Cox PH		
	HR	95% CI	*P*	HR	95% CI	*P*
Age (per one-year increase)	1.04	1.02–1.05	<.001	1.02	1.00–1.04	.010
Sex, male	0.99	0.81–1.22	.94	1.26	0.99–1.60	.058
BMI (per 1 kg/m^2^ increase)	1.05	1.04–1.07	<.001	1.03	1.01–1.05	<.001
History of AF	2.48	1.97–3.12	<.001	2.19	1.73–2.77	<.001
History of diabetes mellitus	2.41	1.98–2.94	<.001	1.88	1.52–2.32	<.001
History of PAD	2.66	1.94–3.66	<.001	1.67	1.20–2.33	.002
Time between MI and baseline (per one-year increase)	1.06	1.04–1.09	<.001	1.04	1.02–1.07	<.001
Smoking						
Never	Reference	—	—	Reference	—	—
Former	1.38	1.14–1.68	.001	1.14	0.93–1.38	.21
Current	1.86	1.46–2.38	<.001	1.64	1.26–2.12	<.001
*Biomarkers (per 1 SD increase)*						
Calcium, mmol/L	0.89	0.82–0.96	.005	0.90	0.83–0.98	.014
Creatinine, µmol/L	1.12	1.08–1.17	<.001	0.79	0.69–0.89	<.001
Cystatin C, mg/L	1.28	1.23–1.32	<.001	1.50	1.32–1.69	<.001
GGT, U/L	1.13	1.07–1.19	<.001	1.09	1.03–1.16	.004
Haemoglobin, g/dL	0.82	0.76–0.89	<.001	0.85	0.78–0.93	<.001
Mean reticulocyte volume, fL	1.27	1.17–1.38	<.001	1.14	1.05–1.24	.002
Neutrophil percentage, %	1.14	1.05–1.24	.002	1.14	1.05–1.24	.003
Monocyte count, 10^9^ cells/L	1.11	1.07–1.15	<.001	1.12	1.06–1.18	<.001

Neutrophil percentage represents the proportion of neutrophils among total leukocytes.

AF, atrial fibrillation; CI, confidence interval; GGT, gamma glutamyl transferase; HR, hazard ratio; PAD, peripheral artery disease; PH, proportional hazards; STEMI, ST-elevation myocardial infarction.

Age, BMI, current smoking status and time between occurrence of MI and study baseline inclusion, were associated with an increased risk of HF. A medical history of AF, diabetes mellitus, or PAD was also associated with an increased risk of HF. Out of the explored biomarkers, increased risk of HF was found for cystatin-C, mean reticulocyte volume, neutrophil percentage, monocyte count, and gamma glutamyl transferase (GGT), while lowered risk was found for calcium, creatinine, and haemoglobin. Male sex was not significantly associated with increased HF risk.

After treating ACM as a competing risk for incident HF, we found that age, BMI, time between occurrence of MI and study baseline, current smoking, cystatin C, haemoglobin, mean reticulocyte volume, neutrophil percentage, monocyte count, and a history of AF or diabetes mellitus, remained significantly associated with incident HF post-MI (*Table 3*). Conversely, associations of calcium, creatinine, GGT, and a history of PAD lost statistical significance. Effect sizes remained stable for the remaining predictors.

**Table 3:** Associations between baseline risk factors and incident HF, with all-cause mortality as a competing risk

Variable	Multivariable competing risk regression^a^		
	sHR	95% CI	*P*
Age (per one-year increase)	1.02	1.00–1.03	.037
Sex, male	1.10	0.85–1.42	.48
BMI, kg/m^2^	1.03	1.01–1.05	<.001
History of AF	2.07	1.61–2.67	<.001
History of diabetes mellitus	1.65	1.31–2.07	<.001
History of PAD	1.41	0.98–2.02	.065
Time between MI and baseline (per one-year increase)	1.04	1.01–1.07	.004
Smoking			
Never	Reference	—	—
Former	1.11	0.91–1.36	.30
Current	1.53	1.17–1.99	.002
*Biomarkers (per 1 SD increase)*			
Calcium, mmol/L	0.92	0.84–1.00	.064
Creatinine, µmol/L	0.85	0.71–1.01	.065
Cystatin C, mg/L	1.33	1.14–1.55	<.001
GGT, U/L	1.05	0.99–1.12	.12
Haemoglobin, g/dL	0.88	0.79–0.96	.007
Mean reticulocyte volume, fL	1.13	1.03–1.23	.009
Neutrophil percentage, %	1.12	1.03–1.21	.009
Monocyte count, 10^9^ cells/L	1.11	1.06–1.16	<.001

Neutrophil percentage represents the proportion of neutrophils among total leukocytes.

AF, atrial fibrillation; CI, confidence interval; GGT, gamma glutamyl transferase; PAD, peripheral artery disease; sHR, subdistribution hazard ratio; STEMI, ST-elevation myocardial infarction.

^a^Based on the Fine-Gray model.

### Model performance

The C-indices of the final model as observed in the training cohort, 5-fold cross-validation sets and the hold-out set were 0.70 (95% CI 0.68–0.72), 0.67 (95% CI 0.56–0.78), and 0.67 (95% CI 0.63–0.71), respectively. The optimism-corrected C-index was 0.68 for the final model. Furthermore, our model showed modest discrimination ability for 10-year incident HF risk in men (C-index = 0.70) and women (C-index = 0.72), while the PCP-HF score showed suboptimal discrimination in men (C-index = 0.59) and women (C-index = 0.59) (Supplementary Table S5).

**Figure 5:**
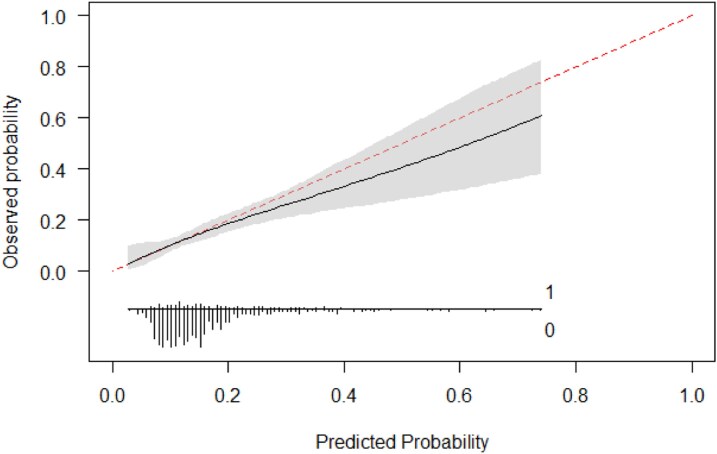
The continuous line denotes model calibration; the dashed line represents perfect calibration, and the dark fields show 95% confidence intervals for model calibration

*Figure 5* shows the recalibrated calibration curve of the predicted and observed HF risk probabilities at 10 years. Numerically, the final model showed agreement between observed and predicted risk (GB-test, *P* = .328). Overall, model calibration was adequate, with appropriate model calibration for the majority (88.1%) of subjects who had a predicted 10-year risk probability below 25%, and slight risk overestimation for participants with predicted risk probabilities above the 25% threshold. Despite this slight overestimation, CI of the calibration curve fully included the perfect calibration diagonal. Further numeric calibration details are shown in Supplementary Table S6. NRI assessment revealed that our final Cox model improved risk category classification in our train set by 23.0% (12.9%–29.2%), compared with a Cox model that included variables used in the PCP-HF score (Supplementary Table S7).

## Discussion

In this study, we identified multiple clinical and biochemical predictors of incident HF following myocardial infarction, including age, BMI, smoking, AF, diabetes, and markers of renal and haematologic function. Sex was not significantly associated with increased HF risk. Women with STEMI exhibited a higher cumulative incidence of HF than those with NSTEMI. The prediction model showed moderate discrimination across all cohorts, adequate calibration for low-risk individuals, and slightly overestimated risk for high-risk individuals.

### Incidence of HF following MI

While most previous studies have assessed the incidence of HF shortly after MI, few have examined the long-term HF incidence after MI. We report an HF incidence of 14.7 per 1000 person-years over the entire follow-up of 15.5 years, which is notably lower than the 64 per 1000 person-years reported over 3.7 years of follow-up in a prior nationwide cohort of MI patients in England (CALIBER).^13^ Similarly, a nationwide analysis in the CVDNOR project reported higher HF incidence rates of 31 per 1000 person-years for men and 46 per 1000 person-years for women during 3.2 years of follow-up.^3^ Discrepancies in incidence rates may be explained by differences in study population characteristics, such as age, prevalence of comorbidities; including diabetes mellitus and stroke, and follow-up duration, compared with prior cohorts. Moreover, improvements in revascularization techniques, such as the use of percutaneous coronary intervention (PCI), decrease infarct size, thereby lowering the risk of HF development.^14^ Furthermore, the increased use of secondary prevention measures, including medical therapies, reduce adverse left ventricular remodelling and subsequent HF risk.^15,16^ Nevertheless, our incidence rates may be underestimated due to survivorship bias, as individuals who developed HF prior to study entry were excluded.

### Sex and MI type

To our knowledge, this is the first study to assess whether HF incidence varies both by MI subtype and by sex. Our study found no significant differences in HF incidence rates by sex or by MI subtype (NSTEMI vs STEMI). When sex and MI subtypes were combined, higher incidence of HF was found in women with STEMI, compared with women with NSTEMI, although this difference was at the threshold of statistical significance (*P* = .05). This could be partially explained by the fact that individuals with STEMI had longer times between MI diagnosis and study baseline, compared with individuals with NSTEMI; although it is unclear why we did not find a similar pattern in men. A previous study has observed a higher incidence of HF in STEMI women than STEMI men.^17^ Another study reported that women were more likely to develop HF than men, regardless of MI type.^18^ Our results also suggest that men with NSTEMI had higher incidence of HF than women with NSTEMI (*P* = .06). These findings are in line with a previous study, which found suggestive sex-differences of acute HF incidence in NSTEMI individuals (*P* = .071). Finally, our study revealed a trend towards an interaction between sex and MI subtype (*P* = .095).

Overall, our study provides modest evidence for sex-MI subtype interactions in HF incidence. However, because our study population consisted primarily of men (81.4%), our analyses likely suffered from reduced statistical power for women-specific and sex comparison analyses. Future studies should attempt to replicate these findings in studies on patients with MI that have comparable representation of both MI-subtypes and sexes.

### Predictors of incident HF following MI

Most of the identified predictors are well-established and align with previous research in patients with MI.^8,19^ Moreover, the majority of identified risk factors, excluding blood count variables, are in line with HF risk factors found in general population cohorts free of prevalent MI.^20,21^ However, our findings also indicate a possible role for monocyte count and neutrophil percentage, which are less frequently reported but are implicated in HF risk. After accounting for competing risk by all-cause mortality, calcium, creatinine, GGT, and a history of PAD lost statistical significance, suggesting that their associations may be more linked to mortality rather than to HF. Therefore, these predictors should be interpreted with caution. Conversely, the eleven predictors that remained significant are more clearly linked to incident HF.

In line with previous research, we found that elevated monocyte count and neutrophil percentage were independently associated with incident HF following MI.^22^ Monocyte count has been found to distinguish individuals with severe HF (Killip class III-IV) from those with non-severe HF (Killip class I-II), as well as predict the development of severe HF after MI.^22^ Monocytes are central to the pathophysiology of MI, where they are activated upon myocardial ischaemia to mediate both inflammation and tissue repair by recruiting cytokines and chemokines.^23^ However, disturbances in monocytes activity may contribute to the progression from MI to HF.^24^ Similarly, while neutrophils are essential for clearing dead cells, they can exacerbate myocardial injury by releasing proteolytic enzymes and pro-inflammatory mediators, which triggers adverse remodelling that can result in HF.^25^ Furthermore, neutrophil count was previously identified as an independent predictor for HF with reduced ejection fraction (HFrEF) in patients with prevalent STEMI.^26^ Finally, haemoglobin and mean reticulocyte volume, both markers of anaemia,^27,28^ were independently associated with incident HF. Anaemia, subsequently, has been defined as an important risk-factor for incident HF in STEMI patients.^29^

### Model performance

Overall, the discriminative ability of our model was modest, with an optimism-corrected train set C-index of 0.68 and a holdout-set C-index of 0.67. While modest, these performances align with findings from a large population-based cohort study of MI individuals without a history of HF, which demonstrated modest discrimination (C-index = 0.64) for incident HF.^30^ To date, few studies have examined incident HF post-MI with comparable endpoints and populations, as most prognostic models focus on HF hospitalization or mortality, limiting direct comparison with our results.^31–33^ Discriminative ability in our study was higher than that of the PCP-HF score, which showed suboptimal performance. This suboptimal performance may be because the PCP-HF score was not designed for a post-MI setting; in our previous work on individuals without history of MI, performance was better, with C-indices up to 0.77.^34^

Model calibration was found to be adequate through both numerical and visual assessment. While the model appeared to slightly overestimate HF risk, the perfect calibration diagonal fully fit within the confidence interval of the calibration curve. This slight overestimation could potentially be caused by overfitting, despite our recalibration efforts. NRI showed a meaningful improvement for the final model, compared with the model derived from the PCP-HF score. Since uniform cut-off points for risk percentages specific to post-MI incident HF have not yet been established, we chose cut-off points based on pragmatic considerations for calculating NRI.

Altogether, model performance was adequate, although clinical applicability warrants caution. Further model refinement, such as the inclusion of (angiographic) MI characteristics, is needed to improve clinical potential.

### Clinical implications

Our study extends the current knowledge on predictors of incident HF following MI by highlighting the potential of various blood count biomarkers. These less established biomarkers may support risk stratification and early identification of individuals at increased risk of developing HF after MI. Furthermore, our final model consists of non-invasive risk factors that are easily measured (i.e. patient characteristics, medical history data, routine laboratory values), making it suitable for use in clinical practice.

Our results provide valuable insights into the interplay between HF and MI in the general population. Proper assessment of the implicated risk factors in clinical practice may enable early identification of high-risk individuals and guide treatment decisions or preventive measures to reduce HF development after MI. Individuals at high risk of HF, particularly those with a history of AF, diabetes mellitus, or PAD, could benefit from pharmacotherapy. For instance, glucagon-like peptide-1 (GLP-1) receptor agonists have been shown to reduce HF hospitalization in diabetic patients.^35^ Additionally, lifestyle modifications, including smoking cessation, body weight regulation, optimization of blood pressure, and glycaemic control, remain crucial for lowering HF risk and improving overall cardiovascular outcomes.^36^

While MI remains an important precursor to HF development, its relevance may be overshadowed by other risk factors. A systematic review,^37^ which identified HF risk prediction models published between 1990 and 2016, identified 40 incident HF risk scores, of which 14 included MI as a predictor. Of these 14 models, the highest performing (C-index >0.80) models were derived from community-based cohort studies,^38,39^ although these studies lacked any assessment of calibration or validation. Conversely, models with some form of calibration or validation, showed adequate performances in diabetic patients^40^ (C-index = 0.751) and patients with AF (C-index = 0.71).^41^ Moreover, a recent systematic review^42^ of 238 HF risk models in general populations, identified the Predicting Risk of CVD EVENTs (PREVENT), Atherosclerosis Risk in Communities (ARIC), and Multi-Ethnic Study of Atherosclerosis (MESA) models as the most promising HF risk prediction models, deserving of further validation and clinical implementation in the future. Notably, none of these models consider history of MI as a risk-factor, although MESA did include family history of MI.

Aforementioned models were constructed in community-based studies. Models predicting incident HF in individuals with history of MI are scarce. A heart failure risk model for patients with prevalent MI or unstable angina (LIPID-heart failure risk model) showed adequate performance when both clinical data and biomarkers were used (C-index = 0.77).^43^ In this context, our study contributes to the cumulative knowledge of incident HF risk by specifically focusing on incident HF risk in MI patients. A recent systematic review of risk prediction models for HF after MI^44^ published up to June 1st 2023, only found studies performed in China, and reported high variability in model performances (C-indices between 0.60 and 0.90), but also reported that included studies were hindered by a general lack of model calibration and validation, a high risk of bias, and a large heterogeneity in study population characteristics. So far, there have been no similar systematic reviews published on European and American study-based incident HF risk prediction models in MI patients.

Overall, our models provide modest prognostic potential for incident HF in MI patients, using non-invasive characteristics. Nevertheless, because model performance was only modest, future clinical research should explore the potential prognostic value of risk factors absent from our study, such as ECG-metrics and (angiographic) MI characteristics; and confirm our findings in other large population-based cohorts.

### Strengths and limitations

Our study has several strengths and limitations. The UK Biobank is well-established and clearly documented, providing us with a large cohort of MI individuals in the general population, enabling in-depth evaluation of incident HF. Our study is also strengthened by rigorous cross-validation and hold-out set validation used to assess model performances.

A limitation of our study is that we were unable to categorize HF based on HF subtypes [HFrEF vs HF with preserved ejection (HFpEF)] or New York Heart Association (NYHA) class, limiting our study’s ability to address HF subtype-specific risk profiles. Another limitation is the absence of N-terminal pro-B-type natriuretic peptide (NT-proBNP) and cardiac troponin, as these are not always measured in general population cohorts. Since NT-proBNP is a strong indicator of HF severity and prognosis,^45^ its inclusion would likely have improved model performance. Another limitation is that our primary endpoint, incident HF, was based on the first available in-hospital diagnosis, which could have resulted in under-ascertainment of outpatient HF.

Furthermore, data pertaining to MI characteristics, such as infarct size and location, was unavailable; this data could have strengthened our findings by expanding on MI properties, since larger infarcts and anterior location are linked to greater HF risk.^46,47^ Likewise, because MI was similarly defined as first-time in-hospital diagnosis, the impact of recurrent MI was unaccounted for.^8^ The UK Biobank population also suffers from a ‘healthy’ participant bias^48^ and is predominantly white, limiting our ability to extrapolate these findings to other populations. Also, because we excluded patients who developed HF before baseline, we may have introduced survivorship bias. Furthermore, our study population was predominantly male and thus may have limited power for sex-stratified analyses and generalizability to female post-MI patients. Despite this, we still noted a higher STEMI-related HF risk than NSTEMI-related HF risk in women. Furthermore, while our prediction model has undergone rigorous internal validation, our model lacks external validation which limits the generalizability of our model. Finally, we could not examine effects of time-dependent variables (i.e. temporal biomarker trajectories, changes in medication use) on incident HF risk.

## Conclusion

In this study we developed and validated a risk prediction model for incident HF following MI in the general population. We identified 11 independent predictors for incident HF after MI, of which various blood count variables were most novel. Our model showed modest discrimination and adequate calibration, with sufficient internal validation. Future studies should aim to externally validate the model in more fairly sex-balanced datasets and evaluate the impact of preventive strategies, including pharmacological and lifestyle interventions, on lowering HF risk after MI.

## Supplementary Material

xvag187_Supplementary_Data

## Declarations

### Data Availability

Access to the data can be requested directly at the UK Biobank, via the route as installed by the UK Biobank.

### Pre-registered Clinical Trial Number

None supplied.
