# Supplementary material for: Prediction of incident heart failure in men and women with a history of myocardial infarction
Source: ESC Heart Fail. 2026 Jun 30;13(4):xvag187. doi: 10.1093/eschf/xvag187 (PMC13395088; doi:10.1093/eschf/xvag187)
Supplement: xvag187_Supplementary_Data [file xvag187_supplementary_data.docx]

**SUPPLEMENTARY INFORMATION**

**Table S1.** *ICD-10* diagnosis codes.

| Diagnosis | *ICD-10* codes |
| --- | --- |
| Myocardial infarction |  |
| STEMI | I21.0, I21.1, I21.2, I21.3, I22.0, I22.1, I22.8 |
| NSTEMI | I21.4, I21.9, I22.9 |
| Atrial fibrillation/flutter | I48 |
| Heart failure | I50 |
| Stroke | I60 – I64 |
| Peripheral artery disease | I70.0, I70.1, I70.2, I73 |
| Malignant cancer |  |
| Colorectal cancer | C18 – C20 |
| Lung cancer | C34 |
| Breast cancer | C50 |
| Haematological cancer | C81 – C96 |
| Diabetes mellitus type 2 | E11 |
| Obesity | E66 |
| Disorders of lipoprotein metabolism | E78 |
| Chronic kidney disease | N18 |

**Legend:** List of diagnosis codes used to extract medical history variables.

**Table S2.** *List of explored predictor variables and their respective field ID codes*.

| Variable | *Field ID code* |
| --- | --- |
| Age | P21003_i0 |
| Sex | P31 |
| Ethnic background | P21000_i0 |
| Employment status | P6142_i0 |
| BMI (kg/m^2^) | P21001_i0 |
| Smoking status | P20116_i0 |
| Alcohol intake frequency | P1558_i0 |
| Systolic blood pressure (mmHg) | P4080_i0_a0 |
| IPAQ activity | P22032_i0 |
| Medication for cholesterol, blood pressure, diabetes, or take exogenous hormones, women only | P6153_i0 |
| Medication for cholesterol, blood pressure, diabetes, or take exogenous hormones, men only | P6177_i0 |
| Blood count biomarkers |  |
| Basophil count (10^9^ cells/L) | P30160_i0 |
| Basophile percentage (%) | P30220_i0 |
| Eosinophil count (10^9^ cells/L) | P30150_i0 |
| Eosinophil percentage (%) | P30210_i0 |
| Haematocrit percentage (%) | P30030_i0 |
| Haemoglobin concentration (g/dL) | P30020_i0 |
| High light scatter reticulocyte count (10^12^ cells/L) | P30300_i0 |
| High light scatter reticulocyte percentage (%) | P30290_i0 |
| Immature reticulocyte fraction (-) | P30280_i0 |
| Lymphocyte count (10^9^ cells/L) | P30120_i0 |
| Lymphocyte percentage (%) | P30180_i0 |
| Mean corpuscular haemoglobin (10^-12^ g) | P30050_i0 |
| Mean corpuscular haemoglobin concentration (g/dL) | P30060_i0 |
| Mean corpuscular volume (10^-15^ L) | P30040_i0 |
| Mean platelet volume (10^-15^ L) | P30100_i0 |
| Mean reticulocyte volume (10^-15^ L) | P30260_i0 |
| Mean sphered cell volume (10^-15^ L) | P30270_i0 |
| Monocyte count (10^9^ cells/L) | P30130_i0 |
| Monocyte percentage (%) | P30190_i0 |
| Neutrophil count (10^9^ cells/L) | P30140_i0 |
| Neutrophil percentage (%) | P30200_i0 |
| Nucleated red blood cell count (10^9^ cells/L) | P30170_i0 |
| Nucleated red blood cell percentage (%) | P30230_i0 |
| Platelet count (10^9^ cells/L) | P30080_i0 |
| Platelet crit (%) | P30090_i0 |
| Platelet distribution width (%) | P30110_i0 |
| Red blood cell count (10^12^ cells/L) | P30010_i0 |
| Red blood cell distribution width (%) | P30070_i0 |
| Reticulocyte count (10^12^ cells/L) | P30250_i0 |
| Reticulocyte percentage (%) | P30240_i0 |
| White blood cell count (10^9^ cells/L) | P30000_i0 |
| Blood biochemistry biomarkers |  |
| Alanine aminotransferase (U/L) | P30620_i0 |
| Albumin (g/L) | P30600_i0 |
| Alkaline phosphatase (U/L) | P30610_i0 |
| Apoliprotein A (g/L) | P30630_i0 |
| Apoliprotein B (g/L) | P30640_i0 |
| Aspartate aminotransferase (U/L) | P30650_i0 |
| C-reactive protein (mg/L) | P30710_i0 |
| Calcium (mmol/L) | P30680_i0 |
| Cholesterol (mmol/L) | P30690_i0 |
| Creatinine (umol/L) | P30700_i0 |
| Cystatin C (mg/L) | P30720_i0 |
| Direct bilirubin (umol/L) | P30660_i0 |
| Gamma glutamyltransferase (U/L) | P30730_i0 |
| Glucose (mmol/L) | P30740_i0 |
| Glycated haemoglobin (HbA1c) (mmol/mol) | P30750_i0 |
| HDL cholesterol (mmol/L) | P30760_i0 |
| IGF-1 (nmol/L) | P30770_i0 |
| LDL cholesterol (mmol/L) | P30780_i0 |
| Lipoprotein A (nmol/L) | P30790_i0 |
| Phosphate (mmol/L) | P30810_i0 |
| SHBG (nmol/L) | P30830_i0 |
| Testosterone (nmol/L) | P30850_i0 |
| Total bilirubin (umol/L) | P30840_i0 |
| Total protein (g/L) | P30860_i0 |
| Triglycerides (mmol/L) | P30870_i0 |
| Urate (umol/L) | P30880_i0 |
| Urea (mmol/L) | P30670_i0 |
| Vitamin D (nmol/L) | P30890_i0 |

**Legend:** List of explored predictor variables and their respective field ID codes. Field ID codes are the codes in which data in stored in the UK Biobank dataset.

**Table S3.** *List of explored predictor variables and their overall missingness*.

| Variable | Missingness (n(%)) |
| --- | --- |
| Age | 0 |
| Sex | 0 |
| Ethnic background | 28 (0.59) |
| Employment status | 50 (1.05) |
| BMI (kg/m^2^) | 42 (0.89) |
| Smoking status | 38 (0.80) |
| Alcohol intake frequency | 16 (0.34) |
| Systolic blood pressure (mmHg) | 287 (6.05) |
| IPAQ activity | 961 (20.3) |
| Medication for cholesterol, blood pressure, diabetes, or take exogenous hormones, women only | 7 (0.79) |
| Medication for cholesterol, blood pressure, diabetes, or take exogenous hormones, men only | 31 (0.80) |
| Blood count biomarkers |  |
| Basophil count (10^9^ cells/L) | 201 (4.24) |
| Basophile percentage (%) | 201 (4.24) |
| Eosinophil count (10^9^ cells/L) | 201 (4.24) |
| Eosinophil percentage (%) | 201 (4.24) |
| Haematocrit percentage (%) | 198 (4.17) |
| Haemoglobin concentration (g/dL) | 198 (4.17) |
| High light scatter reticulocyte count (10^12^ cells/L) | 259 (5.46) |
| High light scatter reticulocyte percentage (%) | 259 (5.46) |
| Immature reticulocyte fraction (-) | 259 (5.46) |
| Lymphocyte count (10^9^ cells/L) | 201 (4.24) |
| Lymphocyte percentage (%) | 201 (4.24) |
| Mean corpuscular haemoglobin (10^-12^ g) | 198 (4.17) |
| Mean corpuscular haemoglobin concentration (g/dL) | 198 (4.17) |
| Mean corpuscular volume (10^-15^ L) | 198 (4.17) |
| Mean platelet volume (10^-15^ L) | 198 (4.17) |
| Mean reticulocyte volume (10^-15^ L) | 259 (5.46) |
| Mean sphered cell volume (10^-15^ L) | 259 (5.46) |
| Monocyte count (10^9^ cells/L) | 201 (4.24) |
| Monocyte percentage (%) | 201 (4.24) |
| Neutrophil count (10^9^ cells/L) | 201 (4.24) |
| Neutrophil percentage (%) | 201 (4.24) |
| Nucleated red blood cell count (10^9^ cells/L) | 201 (4.24) |
| Nucleated red blood cell percentage (%) | 201 (4.24) |
| Platelet count (10^9^ cells/L) | 198 (4.17) |
| Platelet crit (%) | 198 (4.17) |
| Platelet distribution width (%) | 198 (4.17) |
| Red blood cell count (10^12^ cells/L) | 198 (4.17) |
| Red blood cell distribution width (%) | 198 (4.17) |
| Reticulocyte count (10^12^ cells/L) | 259 (5.46) |
| Reticulocyte percentage (%) | 259 (5.46) |
| White blood cell count (10^9^ cells/L) | 198 (4.17) |
| Blood biochemistry biomarkers |  |
| Alanine aminotransferase (U/L) | 310 (6.54) |
| Albumin (g/L) | 655 (13.8) |
| Alkaline phosphatase (U/L) | 308 (6.49) |
| Apoliprotein A (g/L) | 657 (13.9) |
| Apoliprotein B (g/L) | 336 (7.08) |
| Aspartate aminotransferase (U/L) | 329 (6.94) |
| C-reactive protein (mg/L) | 320 (6.75) |
| Calcium (mmol/L) | 657 (13.9) |
| Cholesterol (mmol/L) | 307 (6.47) |
| Creatinine (umol/L) | 310 (6.54) |
| Cystatin C (mg/L) | 309 (6.51) |
| Direct bilirubin (umol/L) | 594 (12.5) |
| Gamma glutamyltransferase (U/L) | 313 (6.60) |
| Glucose (mmol/L) | 656 (13.8) |
| Glycated haemoglobin (HbA1c) (mmol/mol) | 342 (7.21) |
| HDL cholesterol (mmol/L) | 656 (13.8) |
| IGF-1 (nmol/L) | 328 (6.92) |
| LDL cholesterol (mmol/L) | 316 (6.66) |
| Lipoprotein A (nmol/L) | 1409 (29.7) |
| Phosphate (mmol/L) | 667 (14.1) |
| SHBG (nmol/L) | 682 (14.4) |
| Testosterone (nmol/L) | 552 (11.6) |
| Total bilirubin (umol/L) | 329 (6.94) |
| Total protein (g/L) | 659 (13.9) |
| Triglycerides (mmol/L) | 313 (6.60) |
| Urate (umol/L) | 312 (6.58) |
| Urea (mmol/L) | 311 (6.56) |
| Vitamin D (nmol/L) | 355 (7.48) |

**Legend:** List of explored predictor variables and their respective missingness. Missingness is defined in absolute values and percentages.

**Table S4.** *Baseline characteristics stratified by type of MI.*

|  |  |  | **Type of MI** | |  |
| --- | --- | --- | --- | --- | --- |
|  | **Valid cases** | **All, n=4,743** | **NSTEMI, n=2,130** | **STEMI, n=2,613** | ***P* value** |
| ***Demographic characteristics*** |  |  |  |  |  |
| **Age, y** | 4,743 (100%) | 62 (58–66) | 63 (58–66) | 62 (58–66) | 0.067 |
| **Sex, male** | 4,743 (100%) | 3,860 (81.4%) | 1,670 (78.4%) | 2,190 (83.8%) | <0.001 |
| **Ethnicity** | 4,715 (99%) |  |  |  | 0.11 |
| White |  | 4,459 (94.6%) | 1,989 (94.0%) | 2,470 (95.1%) |  |
| Asian or Asian British |  | 168 (3.6%) | 78 (3.7%) | 90 (3.5%) |  |
| Black or Black British |  | 32 (0.7%) | 20 (0.9%) | 12 (0.5%) |  |
| Mixed/other |  | 56 (1.2%) | 30 (1.4%) | 26 (1.0%) |  |
| **Body mass index, kg/m^2^** | 4,701 (99%) | 28.4 (25.9–31.5) | 28.4 (25.8–31.6) | 28.4 (25.9–31.5) | 0.84 |
| **Systolic blood pressure, mm Hg** | 4,456 (94%) | 135 (124–149) | 136 (124–150) | 135 (123–149) | 0.21 |
| **Time between MI and baseline, years** | 4,743 (100%) | 4.9 (2.3–7.9) | 3.9 (1.8–6.9) | 5.7 (3.0–8.5) | <0.001 |
| ***Medical history*** |  |  |  |  |  |
| **Atrial fibrillation** | 4,743 (100%) | 355 (7.5%) | 190 (8.9%) | 165 (6.3%) | <0.001 |
| **Chronic kidney disease** | 4,743 (100%) | 27 (0.6%) | 15 (0.7%) | 12 (0.5%) | 0.26 |
| **Diabetes mellitus** | 4,743 (100%) | 554 (11.7%) | 270 (12.7%) | 284 (10.9%) | 0.054 |
| **Dyslipidaemias** | 4,743 (100%) | 2,372 (50.0%) | 1,076 (50.5%) | 1,296 (49.6%) | 0.53 |
| **Obesity** | 4,743 (100%) | 154 (3.2%) | 74 (3.5%) | 80 (3.1%) | 0.43 |
| **Peripheral artery disease** | 4,743 (100%) | 175 (3.7%) | 73 (3.4%) | 102 (3.9%) | 0.39 |
| **Hypertension** | 4,743 (100%) | 2,231 (47.0%) | 1,049 (49.2%) | 1,182 (45.2%) | 0.006 |
| **Stroke** | 4,743 (100%) | 91 (1.9%) | 52 (2.4%) | 39 (1.5%) | 0.018 |
| **Cancer** | 4,743 (100%) |  |  |  |  |
| Breast cancer |  | 29 (0.6%) | 16 (0.8%) | 13 (0.5%) | 0.26 |
| Colorectal cancer |  | 27 (0.6%) | 12 (0.6%) | 15 (0.6%) | 0.96 |
| Haematologic cancer |  | 31 (0.7%) | 17 (0.8%) | 14 (0.5%) | 0.26 |
| Lung cancer |  | 5 (0.1%) | 3 (0.1%) | 2 (0.1%) | 0.66 |
| ***Lifestyle factors*** |  |  |  |  |  |
| **Alcohol intake frequency** | 4,727 (100%) |  |  |  | 0.13 |
| Daily or almost daily |  | 965 (20.4%) | 440 (20.7%) | 525 (20.1%) |  |
| Three or four times a week |  | 1,085 (23.0%) | 487 (23.0%) | 598 (22.9%) |  |
| Once or twice a week |  | 1,165 (24.6%) | 544 (25.6%) | 621 (23.8%) |  |
| One to three times a month |  | 547 (11.6%) | 255 (12.0%) | 292 (11.2%) |  |
| Special occasions only |  | 575 (12.2%) | 233 (11.0%) | 342 (13.1%) |  |
| Never |  | 390 (8.3%) | 162 (7.6%) | 228 (8.7%) |  |
| **Smoking** | 4,705 (99%) |  |  |  | <0.001 |
| Never |  | 1,534 (32.6%) | 762 (36.0%) | 772 (29.9%) |  |
| Former |  | 2,490 (52.9%) | 1,042 (49.2%) | 1,448 (56.0%) |  |
| Current |  | 681 (14.5%) | 315 (14.9%) | 366 (14.2%) |  |
| **Physical activity** | 3,782 (80%) |  |  |  | 0.50 |
| Low |  | 800 (21.2%) | 375 (22.0%) | 425 (20.5%) |  |
| Moderate |  | 1,506 (39.8%) | 677 (39.7%) | 829 (39.9%) |  |
| High |  | 1,476 (39.0%) | 654 (38.3%) | 822 (39.6%) |  |
| ***Medication use*** | 4,675 (99%) |  |  |  |  |
| Cholesterol lowering medication |  | 4,400 (92.8%) | 1,945 (91.3%) | 2,455 (94.0%) | <0.001 |
| Blood pressure medication |  | 3,626 (76.4%) | 1,616 (75.9%) | 2,010 (76.9%) | 0.39 |
| Insulin |  | 200 (4.2%) | 94 (4.4%) | 106 (4.1%) | 0.54 |
| Hormone replacement therapy |  | 32 (0.7%) | 19 (0.9%) | 13 (0.5%) | 0.10 |
| Oral contraceptive |  | 5 (0.1%) | 2 (0.1%) | 3 (0.1%) | >0.99 |
| No medication use |  | 115 (2.4%) | 61 (2.9%) | 54 (2.1%) | 0.076 |
| ***Laboratory results*** |  |  |  |  |  |
| **Haemoglobin, g/dL** | 4,545 (96%) | 14.6 (13.8–15.3) | 14.5 (13.7–15.3) | 14.7 (13.8–15.4) | 0.005 |
| **Creatinine, µmol/L** | 4,433 (93%) | 80.6 (70.8–91.4) | 80.1 (70.3–91.5) | 81.1 (71.2–91.4) | 0.18 |
| **Cholesterol, mmol/L** | 4,436 (94%) | 4.2 (3.7–4.8) | 4.2 (3.7–4.8) | 4.2 (3.7–4.8) | 0.34 |
| **Triglycerides, mmol/L** | 4,430 (93%) | 1.6 (1.1–2.3) | 1.6 (1.1–2.3) | 1.7 (1.2–2.3) | 0.053 |
| Values are reported as n (%), otherwise as median (Q1–Q3).  MI indicates myocardial infarction; NSTEMI indicates non-ST-elevation MI; STEMI, ST-elevation MI. | | | | | |

**Table S5.** *Discrimination ability of our model and the PCP-HF risk model.*

| **Study population** | Final Cox model | PCP-HF score model |
| --- | --- | --- |
| White men | 0.70 | 0.59 |
| White women | 0.72 | 0.59 |

**Legend:** 10-year HF risk assessment in the full train dataset. The PCP-HF score describes different formulas for men and women, as well as for white and black individuals. Because our population is predominantly white, we derived performance metrices for white men and white women, separately. Discrimination is defined in C-indices.

**Table S6.** *Numeric details of model calibration*

| **Metric** | **Estimate** | **95% CI** |
| --- | --- | --- |
| E_average | 0.018 | 0.0058 – 0.035 |
| E_50 | 0.0022 | 0.0026 – 0.030 |
| E_90 | 0.0320 | 0.0096 – 0.075 |
| E_max | 0.1308 | 0.0208 – 0.363 |
| ECI | 0.0530 | 0.00058 – 0.323 |

**Legend:** Estimates, with 95% confidence intervals, of various calibration metrics. E_average describes the average difference between prediction and actual probability of incident HF risk to be 0.018 (or 1.8%) with a 95% CI of [0.0058 – 0.035]. E_50 describes the median difference between prediction and observed probabilities (i.e. 50% of all differences are 0.0022 or smaller). E_90 is like E_50, but for the 90^th^ percentile difference; 90% of all differences are 0.032 or smaller. E_max is the largest observed difference. Finally, ECI is the estimated calibration index, which describes the average squared difference between predicted and observed probabilities (multiplied by 100). 95% confidence intervals were calculated via simulation-based inference with 1000 replicates.

**Table S7.** *Reclassification of events and non-events at 10 years of follow-up.*

|  | Predicted risk by study model | | | Individuals with reclassified risk, n(%) | | Net correctly reclassified, n(%) |
| --- | --- | --- | --- | --- | --- | --- |
| Predicted risk by PCP-HF | 0% - 10% | 10 – 20% | > 20% | Increased | Decreased |  |
| Individuals without events (N = 2968) | | | | | | |
| 0% - 10% | 996 | 195 | 20 | 376 (12.6) | 780 (26.3) | 404 (13.6) |
| 10 – 20% | 646 | 708 | 161 |  |  |  |
| > 20% | 20 | 114 | 108 |  |  |  |
| Individuals with events (N = 450) | | | | | | |
| 0% - 10% | 65 | 34 | 5 | 126 (28.0%) | 82 (18.2%) | 44 (9.8) |
| 10 – 20% | 61 | 113 | 87 |  |  |  |
| > 20% | 3 | 18 | 64 |  |  |  |
| Net reclassification improvement (95% CI) 23.0 (12.8 – 29.2) | | | | | | |

**Legend:** Each interior cell contains the number of persons in the corresponding risk categories under the base model (PCP-HF) and the new model (study model). Individuals with higher predicted risk were more likely to be individuals who developed HF. At the same time, individuals with lower predicted risk were more likely to be individuals who remained free of incident HF. Percentages in interior cells are based on the number of non-events or events. Confidence intervals for net reclassification improvement are obtained via internal estimation using bootstrapping with 1000 iterations.
